# Efficacy and Safety of Oral Progestogens (Megestrol Acetate and Medroxyprogesterone Acetate) in Heavily Pretreated Oestrogen Receptor-Positive Metastatic Breast Cancer: A 10-Year Multi-Site Study

**DOI:** 10.3390/cancers18081191

**Published:** 2026-04-08

**Authors:** Iseult M. Browne, Heng Chun Wong, Tazia Irfan, Chloe Chan, Stephen R. D. Johnston, Zoe Kemp, Emma Kipps, Marina Parton, Nicholas C. Turner, Alicia F. C. Okines

**Affiliations:** 1 The Royal Marsden NHS Foundation Trust, London SW3 6JJ, UK; iseult.browne@icr.ac.uk (I.M.B.);; 2 The Institute of Cancer Research, London SW3 6JB, UK

**Keywords:** metastatic breast cancer, oral progestogens, megestrol acetate, medroxyprogesterone acetate

## Abstract

**Background:** Oral progestogens, including megestrol acetate (MA) and medroxyprogesterone acetate (MPA), have largely been superseded by aromatase inhibitors, tamoxifen, and selective oestrogen receptor degraders (SERDs) in oestrogen receptor-positive (ER-positive) metastatic breast cancer. However, they remain an option as late-line therapy after failure of standard treatments. Contemporary data are limited, particularly in patients previously treated with CDK4/6 inhibitors. **Methods:** We conducted a multi-site retrospective analysis of patients with ER-positive metastatic breast cancer treated with MA or MPA between 2014 and 2024 at four hospital sites across London, United Kingdom. Patients were identified using pharmacy dispensing records. Progression-free survival (PFS) and overall survival (OS) were estimated using the Kaplan–Meier method and Cox regression. Subgroup analyses included prior CDK4/6 inhibitor exposure, histology and liver metastases. **Results:** A total of 116 patients were included. Median PFS was 2.4 months (95% CI 2.2–2.9), and median OS was 3.3 months (95% CI 2.7–4.9). Prior CDK4/6 inhibitor exposure was associated with shorter PFS (1.9 vs. 2.8 months; HR 1.59; 95% CI 1.08–2.35, *p* = 0.019) and a trend toward shorter OS (3.1 vs. 3.6 months; HR 1.18, 95% CI 0.80–1.75, *p* = 0.41). Similarly, liver metastases were associated with shorter PFS (2.3 vs. 2.8 months; HR 1.78, 95% CI 1.12–2.85, *p* = 0.015), with a trend toward worse OS (3.1 vs. 4.9 months; HR 1.45, 95% CI 0.93–2.25, *p* = 0.103). A subset of patients derived prolonged benefit, with a 6-month PFS rate of 16%. Toxicity was manageable; thromboembolic events and oedema occurred in 9% and 11% of patients respectively. Appetite improvement was reported in 10%. **Conclusions:** MA and MPA demonstrated modest but clinically relevant late-line activity in heavily pretreated, endocrine-refractory ER-positive metastatic breast cancer. While prior exposure to CDK4/6 inhibitors was associated with shorter PFS, patients without liver metastases appeared to derive the greatest benefit. These findings support a role for oral progestogens in selected patients who have exhausted standard therapeutic options.

## Simple Summary

The oral progestogens megestrol acetate and medroxyprogesterone acetate were previously used in the treatment of metastatic oestrogen receptor-positive breast cancer but have since been superseded by modern agents. They remain an option as late-line therapy and in selected palliative settings. The aim of our retrospective study was to assess the progression-free survival (PFS), overall survival (OS) and toxicity in patients treated with oral progestogens and to determine whether prior CDK4/6 inhibitor exposure affects outcomes. Our results demonstrate that oral progestogens are associated with modest benefits in PFS and OS (Median PFS and OS of 2.4 months and 3.3 months, respectively) with a manageable toxicity profile. Prior CDK4/6 inhibitor exposure and the presence of liver metastases were associated with shorter PFS but not OS. Notably, a subset of patients derived prolonged benefit, with a 6-month PFS rate of 16% and a 12-month PFS rate of 6%. Oral progestogens may thus retain a role as a late-line endocrine option in carefully selected patients.

## 1. Introduction

Endocrine therapy remains the backbone of treatment for oestrogen receptor-positive (ER-positive) metastatic breast cancer. Over recent decades, the therapeutic landscape has evolved, with sequential adoption of tamoxifen, aromatase inhibitors (AIs), and, more recently, targeted combinations incorporating CDK4/6 inhibitors, which now constitute standard first-line therapy for the majority of patients with ER-positive metastatic breast cancer. Intramuscular fulvestrant [1] and oral selective oestrogen receptor degraders (SERDs) given alone [2] or in combination with agents targeting the PIK3CA/AKT-mTOR pathway [3,4] have further expanded endocrine therapy options available. More recently, antibody–drug conjugates have continued to reshape sequencing strategies, resulting in progressively later positioning of older endocrine agents within treatment algorithms [5].

The integration of molecular profiling into routine practice has additionally enabled biomarker-directed treatment strategies, thereby refining therapeutic sequencing and individualising care [6,7]. As a result, the modern management of ER-positive metastatic breast cancer is increasingly characterised by endocrine-based strategies aimed at delaying chemotherapy for as long as clinically feasible. The availability of circulating tumour DNA assays has further facilitated real-time detection of resistance mutations and dynamic monitoring of tumour evolution during treatment, offering the potential to guide therapeutic decision-making as tumour biology evolves [8,9].

Despite these advances, endocrine resistance inevitably develops in most patients with metastatic disease. Mechanisms of resistance are multifactorial and include ligand-independent activation of the oestrogen receptor, *ESR1* mutations, cross-talk with growth factor signalling pathways such as PI3K/AKT/mTOR, dysregulation of cell cycle control via RB pathway alterations, and epigenetic reprogramming [10,11]. The widespread use of CDK4/6 inhibitors in earlier lines of therapy has further altered the biological landscape at progression, with emerging evidence suggesting distinct resistance phenotypes compared with the pre-CDK4/6 era [12,13,14]. Consequently, treatment options in later lines increasingly rely on agents with alternative mechanisms of endocrine modulation or non-cross-resistant strategies. In this context, understanding whether older endocrine agents retain activity after modern targeted therapies is of both clinical and biological interest.

Historically, the oral progestogens megestrol acetate (MA) and medroxyprogesterone acetate (MPA) were widely used in the treatment of advanced breast cancer, typically at doses of 160 mg and 500 mg daily, respectively. Their anti-tumour effects are believed to arise from complex endocrine modulation, including suppression of hypothalamic–pituitary signalling, downregulation of oestrogen receptor expression, and potential direct antiproliferative effects mediated via the progesterone receptor [15,16,17]. However, randomised trials conducted in the late 1980s and early 1990s demonstrated superior progression-free and overall survival with tamoxifen [18] alongside improved tolerability [19], leading to a decline in progestogen use. Subsequent studies established the superiority of AIs such as letrozole and anastrozole over tamoxifen [20,21] and later the benefit of combining endocrine therapy with CDK4/6 inhibitors [22,23,24], forming the current standard of care and further relegating oral progestogens to late-line settings. As therapeutic standards advanced, progestogens were progressively displaced not due to a complete lack of activity but rather relative inferiority compared with newer agents demonstrating improved efficacy and safety profiles.

Despite this, oral progestogens have not disappeared from clinical practice. They continue to be used selectively in heavily pretreated patients [25], in those unfit for chemotherapy, and in resource-limited settings due to their low cost [26], oral administration, and occasional symptomatic benefits such as appetite stimulation in cachexia [27]. In addition, in elderly or frail populations where treatment goals may prioritise symptom control and preservation of quality of life over tumour shrinkage, progestogens may represent a pragmatic therapeutic compromise. Their oral route, minimal requirement for hospital attendance, and relatively predictable toxicity profile can be advantageous in patients with increased vulnerability to treatment toxicity. However, they are associated with clinically relevant toxicities, including weight gain, fluid retention, and an increased risk of thromboembolic events [25]. The thrombotic risk is of particular relevance in the metastatic setting, where patients may already possess multiple pro-thrombotic risk factors, including immobility, visceral disease burden, and prior systemic therapies. Moreover, metabolic effects such as hyperglycaemia and hypertension may be under-recognised in retrospective analyses, further complicating the safety profile. These risks necessitate careful patient selection and clinical vigilance, particularly in older populations with baseline cardiovascular comorbidity.

Notably, there is a paucity of contemporary data evaluating the efficacy and safety of oral progestogens in patients treated within modern therapeutic paradigms, particularly following exposure to CDK4/6 inhibitors. Much of the existing literature predates routine molecular profiling, targeted pathway inhibition, and current standards of endocrine sequencing. Therefore, the relevance of historical efficacy data to present-day practice is uncertain. In particular, it remains unclear whether tumours progressing after CDK4/6 inhibitor-based therapy retain sensitivity to progesterone-mediated endocrine modulation or whether accumulated resistance mechanisms diminish the potential benefit of progestogens. Given the increasingly complex sequencing landscape, real-world evidence examining outcomes in heavily pretreated cohorts is essential to clarify whether progestogens retain a clinically meaningful benefit.

Furthermore, the shifting demographics of metastatic breast cancer with an ageing patient population and increasing survival duration underscore the need to re-evaluate older agents within a contemporary context. As therapeutic lines extend and cumulative toxicities accrue, clinicians are frequently confronted with complex decision-making in late-line disease, balancing modest efficacy, treatment burden, cost, and patient preference. In this setting, real-world data may provide valuable insights into the practical utility of legacy endocrine therapies. Such data are particularly relevant in publicly funded healthcare systems, where cost-effectiveness and equitable access to treatment remain important considerations.

To address this gap, we conducted a 10-year retrospective study to evaluate real-world outcomes associated with MA and MPA in patients with ER-positive metastatic breast cancer treated at The Royal Marsden NHS Foundation Trust (Chelsea, Cavendish Square and Sutton sites) and Kingston Hospital NHS Foundation Trust. Our objectives were to characterise progression-free and overall survival in a modern, heavily pretreated cohort; to explore outcomes in clinically relevant subgroups, including prior CDK4/6 inhibitor exposure and visceral disease; and to comprehensively describe the toxicity profile of oral progestogens in routine practice. By contextualising these findings within the CDK4/6 inhibitor era, we aimed to better define the residual role of progestogens in current therapeutic algorithms. Through this analysis, we sought to inform evidence-based decision-making regarding the continued, selective use of progestogens in late-line ER-positive metastatic breast cancer.

## 2. Materials and Methods

### 2.1. Study Design and Setting

We conducted a multi-site retrospective cohort study over a 10-year period (July 2014 to July 2024) across four hospital sites in London: The Royal Marsden NHS Foundation Trust (Chelsea, Cavendish Square and Sutton sites) and Kingston Hospital NHS Foundation Trust. The study aimed to evaluate the efficacy and safety of oral progestogens, specifically megestrol acetate (MA) and medroxyprogesterone acetate (MPA), in patients with heavily pretreated ER-positive metastatic breast cancer. Patients were identified using local electronic prescribing systems and pharmacy dispensing records.

The study was designed to reflect real-world clinical practice rather than protocol-driven treatment, thereby capturing prescribing patterns, dosing strategies, and outcomes as they occurred in routine care. All participating centres were tertiary referral institutions with dedicated breast oncology services, ensuring consistent standards of systemic therapy delivery and follow-up.

Data extraction was performed using a predefined data collection template to ensure consistency across sites. Where discrepancies were identified between pharmacy records and electronic clinical documentation, case review was undertaken to verify treatment exposure and outcome data.

The study was approved by the institutional review board (protocol code 1470, date of approval 11 February 2025), and patient confidentiality was maintained in accordance with institutional and national guidelines.

### 2.2. Eligibility Criteria

Eligible patients were adult women (≥18 years) with a confirmed diagnosis of ER-positive metastatic breast cancer who received at least one dose of MA or MPA during the study period. Patients were excluded if they were receiving concurrent systemic therapy for a second malignancy or were lost to follow-up while receiving progestogen therapy. Patients with incomplete baseline clinical data or unclear treatment documentation were also excluded from specific analyses. Patients who received progestogens purely for appetite stimulation without documented metastatic disease were not included in this study.

### 2.3. Data Collection

Electronic patient records were reviewed to extract the following variables: demographic data, Eastern Cooperative Oncology Group (ECOG) performance status at the start of progestogen therapy, tumour characteristics (histology, receptor status), metastatic sites, prior systemic therapies including chemotherapy, endocrine therapy, and targeted agents such as CDK4/6 inhibitors, and details of progestogen therapy (drug, dose, start and stop dates, dose modifications).

Outcomes including dates of disease progression, survival status, and adverse events were collected. Adverse events were retrospectively graded according to the Common Terminology Criteria for Adverse Events (CTCAE) version 5.0. Symptomatic improvements, such as appetite stimulation, were also recorded when documented.

### 2.4. Treatment and Dosage

The choice of progestogen, starting dose, and dose escalation strategy was at the discretion of the treating clinician and influenced by drug availability, patient comorbidities, and tolerability. Standard starting doses were MA 160 mg daily and MPA 100–400 mg daily. Dose modifications, interruptions, or discontinuations were recorded, along with reasons for discontinuation, including toxicity or disease progression.

### 2.5. Outcomes

The primary endpoint was progression-free survival (PFS), defined as the time from initiation of oral progestogen therapy to documented disease progression or death from any cause. Secondary endpoints included overall survival (OS), defined as the time from progestogen initiation to death from any cause, and the incidence and severity of treatment-related toxicities.

Exploratory endpoints included the proportion of patients achieving prolonged disease control, defined as remaining progression-free at 6 and 12 months, and subgroup analyses of PFS and OS according to prior CDK4/6 inhibitor exposure, histological subtype (invasive ductal carcinoma versus invasive lobular carcinoma), and the presence of liver metastases. Baseline characteristics of patients achieving prolonged disease control with ≥12 months PFS were additionally described.

### 2.6. Statistical Analysis

Descriptive statistics were used to summarise baseline characteristics, treatment exposure, and toxicities. PFS and OS were estimated using the Kaplan–Meier method, with differences between subgroups assessed using the log-rank test. Hazard ratios (HRs) and 95% confidence intervals (CIs) were calculated using univariable Cox proportional hazards models. Patients who were alive and progression-free at the time of last follow-up were censored. All statistical analyses were performed using R Statistical Software (v4.5.2), and a *p*-value < 0.05 was considered statistically significant.

## 3. Results

### 3.1. Patient Characteristics and Treatment Exposure

A total of 116 female patients were included in our study, with baseline characteristics summarised in Table 1. The median age of patients included was 69 years, with a median ECOG performance status of 2. Seventy-five percent of patients had invasive ductal carcinoma (IDC), while 15% of patients had invasive lobular carcinoma (ILC). Patients received a median of 5 prior lines of treatment for metastatic breast cancer (range 1–11), and 35% had previously received a CDK4/6 inhibitor. Liver metastases were present in 74% of patients.

### 3.2. Survival Results

#### 3.2.1. Progression-Free Survival

**Figure 1:**
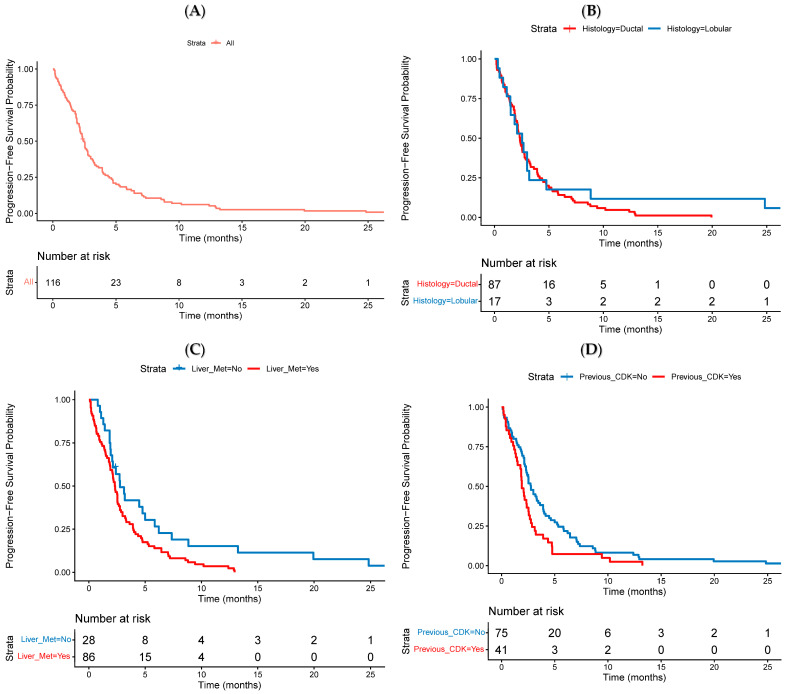
(**A**–**D**): Kaplan–Meier estimates of progression-free survival stratified by clinical factors: (**A**) Entire cohort; (**B**) Tumour histology: ductal vs. lobular; (**C**) Presence vs. absence of liver metastases; (**D**) Prior exposure to CDK4/6 inhibitors.

The median PFS (mPFS) for the entire cohort was 2.4 months (95% CI 2.2–2.9) (Figure 1A). A subset of patients demonstrated prolonged benefit, with 16% (19/116) of patients remaining progression-free beyond 6 months, and 6% (7/116) beyond 12 months. PFS did not differ significantly by histological subtype: patients with IDC had an mPFS of 2.3 months (95% CI 2.1–2.8), while patients with ILC had an mPFS of 2.5 months (95% CI 1.5–4.8; HR 1.28, 95% CI 0.74–2.27, *p* = 0.363) (Figure 1B).

**Figure 2:**
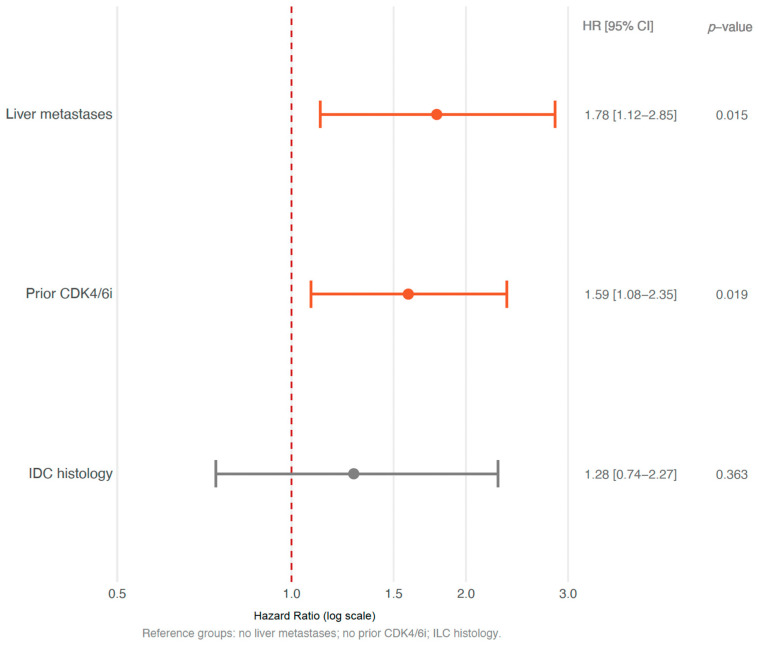
Subgroup analyses of progression-free survival. Forest plot showing hazard ratios with 95% confidence intervals for progression-free survival across prespecified subgroups. The vertical dashed line represents a hazard ratio of 1.0. Orange markers indicate statistically significant comparisons (*p* < 0.05); grey markers indicate non-significant comparisons. IDC, Invasive Ductal Carcinoma; ILC, Invasive Lobular Carcinoma; CDK4/6i, CDK4/6 inhibitor.

Median PFS was significantly shorter in patients with liver metastases, with an mPFS of 2.3 months (95% CI 1.9–2.8) versus 2.8 months (95% CI 2.1–5.9; HR 1.78, 95% CI 1.12–2.85, *p* = 0.015) (Figure 1C). Similarly, prior exposure to CDK4/6 inhibitors was associated with reduced PFS: 1.9 months (95% CI 1.8–2.6) in previously treated patients versus mPFS of 2.8 months (95% CI 2.3–3.9) in those without prior exposure (HR 1.59; 95% CI 1.08–2.35, *p* = 0.019) (Figure 1D). See Figure 2 for a summary of subgroup analyses of PFS.

#### 3.2.2. Overall Survival

**Figure 3:**
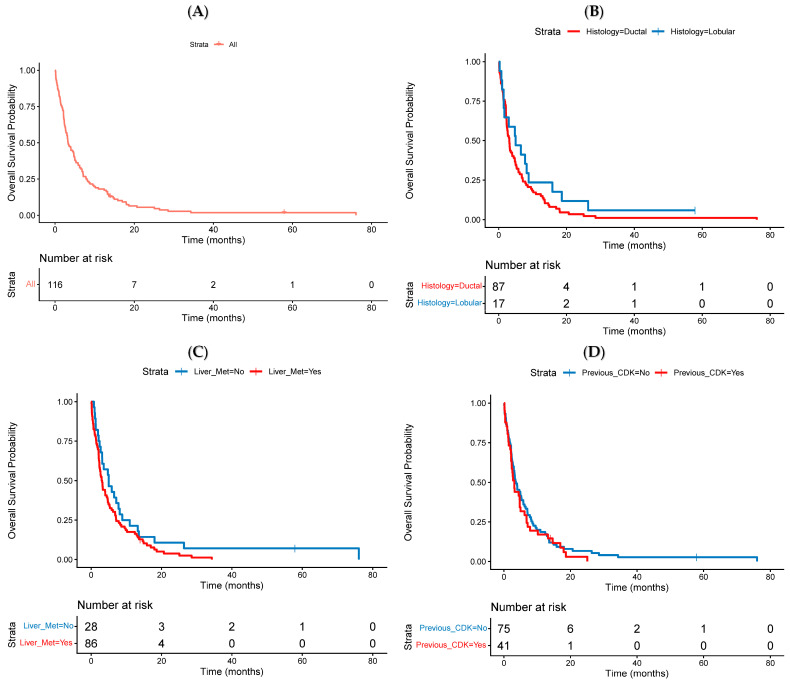
(**A**–**D**): Kaplan–Meier estimates of Overall Survival (OS) stratified by clinical factors: (**A**) Entire cohort; (**B**) Tumour histology: ductal vs. lobular; (**C**) Presence vs. absence of liver metastases; (**D**) Prior exposure to CDK4/6 inhibitors.

The median overall survival (mOS) for the entire cohort was 3.3 months (95% CI 2.7–4.9) (Figure 3A). OS did not differ significantly by histological subtype, although patients with ILC showed a numerically longer mOS of 5.0 months (95% CI 1.7–15.8) compared to 3.1 months (95% CI 2.4–4.7) for patients with IDC (HR 1.45, 95% CI 0.84–2.50, *p* = 0.18) (Figure 3B).

**Figure 4:**
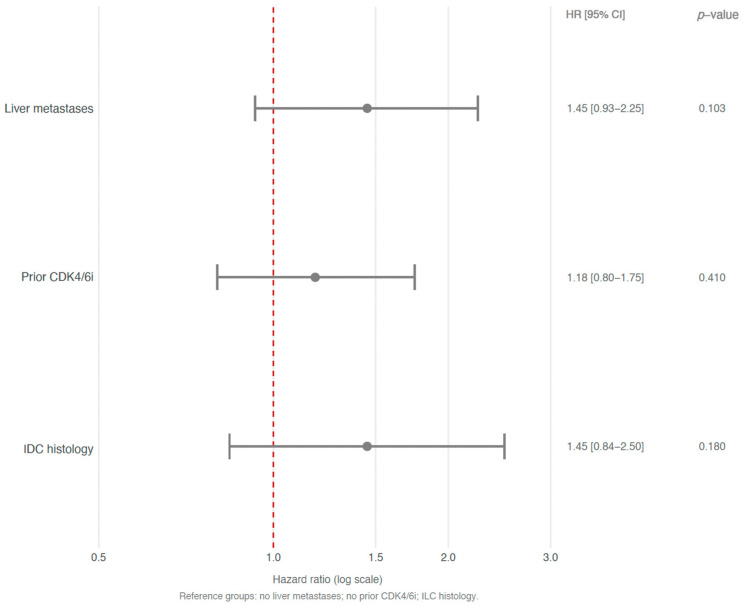
Subgroup analyses of overall survival. Forest plot showing hazard ratios with 95% confidence intervals for overall survival across prespecified subgroups. The vertical dashed line represents a hazard ratio of 1.0. All comparisons were non-significant (*p* ≥ 0.05). IDC, Invasive Ductal Carcinoma; ILC, Invasive Lobular Carcinoma; CDK4/6i, CDK4/6 inhibitor.

Similarly, OS was comparable across other subgroups: patients with liver metastases had an mOS of 3.1 months (95% CI 2.3–4.7) versus 4.9 months (95% CI 3.1–8.8) in those without liver involvement (HR 1.45, 95% CI 0.93–2.25, *p* = 0.103) (Figure 3C). Prior exposure to CDK4/6 inhibitors was associated with a similar OS: 3.1 months (95% CI 2.3–5.0) in exposed patients and 3.6 months (95% CI 2.7–5.8) in those not exposed (HR 1.18, 95% CI 0.80–1.75, *p* = 0.41) (Figure 3D). See Figure 4 for a summary of subgroup analyses of OS.

### 3.3. Patients with Prolonged Disease Control

Seven patients (6%) remained progression-free at 12 months. This group was notably older (median age 78 versus 69 years), had fewer prior lines of therapy, and were less likely to have liver metastases (43% vs. 76%) or prior CDK4/6 inhibitor exposure (14% vs. 37%) compared with the remainder of the cohort. No formal statistical comparisons were performed, and these findings should be considered hypothesis-generating only. Baseline characteristics of this subgroup are summarised in Table 2.

### 3.4. Toxicity

The reported side-effects of oral progestogens are summarised below in Table 3.

During progestogen therapy, seven patients (6%) experienced ≥Grade 3 treatment-emergent thromboembolic events. These included one fatal ischaemic stroke and two cases requiring acute stroke admission. Three patients developed a pulmonary embolism, one of whom required ITU admission for thrombolysis. Additionally, one patient experienced a deep vein thrombosis necessitating hospital admission. Eight patients (7%) discontinued treatment due to intolerance.

Of note, appetite improvement was documented as reported by 11 patients (10%), but this information was not captured consistently across the cohort.

## 4. Discussion

In this 10-year multi-site retrospective analysis, we report the largest contemporary series evaluating the activity of oral progestogens in patients with heavily pretreated ER-positive metastatic breast cancer treated in the CDK4/6 inhibitor era. Our study provides novel real-world insights into outcomes for patients often excluded from prospective trials due to advanced age, frailty, or multiple prior therapies. Our findings suggest that megestrol acetate and medroxyprogesterone acetate confer modest but clinically meaningful disease control in a selected subgroup of patients, with a small proportion experiencing prolonged benefit.

The observed median PFS of 2.4 months and median OS of 3.3 months are shorter than those reported in earlier prospective studies, including the phase II trials by Bines et al., which described a median PFS of 3.9 months and OS of 19.4 months [25]. This difference likely reflects differences in patient selection and treatment sequencing, with our cohort representing a heavily pretreated population, frequently exposed to multiple lines of endocrine therapy, chemotherapy, and targeted agents, including CDK4/6 inhibitors. The high prevalence of liver metastases (74%) and the median of five prior systemic therapy lines highlight the frailty and late-line nature of the population, providing real-world context for the observed modest efficacy. In this context, the modest efficacy observed remains clinically relevant, particularly given the limited therapeutic options available in late-line disease.

When contextualised against other systemic anti-cancer therapies used in late-line ER-positive metastatic breast cancer, the activity of oral progestogens appears broadly comparable. Median PFS with late-line chemotherapy regimens, such as capecitabine or vinorelbine, typically ranges between 2 and 4 months, often accompanied by substantial toxicity [28,29,30]. While newer endocrine agents and antibody–drug conjugates may offer improved outcomes, access, eligibility, toxicity, and patient fitness frequently limit their use. In this context, oral progestogens remain a low-cost oral option that may provide disease stabilisation in selected patients, particularly those unable or unwilling to receive further chemotherapy. The oral route, relative tolerability, and potential symptomatic benefits such as appetite improvement make progestogens a practical option for patients prioritising quality of life over maximal tumour shrinkage.

Patients with prior exposure to CDK4/6 inhibitors had shorter PFS compared with those without such exposure (median 1.9 months versus 2.8 months), indicating a more heavily pretreated and potentially endocrine-resistant population. Despite this, OS was similar between groups (3.1 months versus 3.6 months), suggesting that oral progestogens can still provide disease stabilisation in these patients, even if prior therapy predicts earlier progression. Seven patients (6%) remained progression-free at 12 months, representing a subgroup with prolonged benefit. This group was older (median age 78 versus 69), less likely to have liver metastases (43% versus 76%), and had lower prior exposure to CDK4/6 inhibitors (14% versus 35%), suggesting that careful patient selection may identify those more likely to derive durable benefit.

Similarly, patients with liver metastases experienced shorter PFS (2.3 months versus 2.8 months in patients without liver involvement), while OS remained comparable, highlighting that PFS may not always correlate with OS in this heavily pretreated population. This emphasises that in late-line disease, stabilisation rather than tumour shrinkage may represent clinically meaningful benefit, particularly in terms of avoiding symptomatic progression and maintenance of quality of life.

Toxicity remains a key consideration. While appetite improvement was documented in a minority of patients, the incidence of thromboembolic events was notable, with a proportion being grade 3 or higher. This toxicity profile, alongside rates of peripheral oedema and treatment discontinuation due to intolerance, underscores the need for careful patient selection and monitoring, particularly in older patients and those with a baseline thrombotic risk.

This study has several limitations inherent to its retrospective design, including incomplete toxicity reporting, inconsistent imaging precluding reliable objective response assessment, and heterogeneity in dosing and progestogen selection. Molecular profiling data, including PIK3CA/AKT/PTEN pathway status and *ESR1* mutations, were available only for a minority of patients, as routine molecular testing was not yet established for much of the study period. Treatment decisions were influenced by drug availability rather than protocolised criteria, which may introduce selection bias and limit generalisability. Nonetheless, this study provides valuable real-world data on progestogen use in heavily pretreated, contemporary populations, including those with prior CDK4/6 inhibitor exposure who are often underrepresented in prospective trials.

Taken together, our findings suggest that oral progestogens retain a potential role in carefully selected patients, particularly for those prioritising oral administration, symptom control, or avoidance of more toxic therapies.

## 5. Conclusions

In conclusion, this 10-year retrospective analysis represents one of the largest contemporary real-world evaluations of oral progestogens in ER-positive metastatic breast cancer in the CDK4/6 inhibitor era. Megestrol acetate and medroxyprogesterone acetate demonstrated modest but clinically relevant activity in heavily pretreated patients, with a small subset deriving durable benefit. Outcomes appear less favourable in patients with liver metastases, and thromboembolic risk remains an important consideration.

While newer therapies continue to redefine the treatment landscape, oral progestogens retain a selective role as a late-line endocrine option in carefully selected patients. Prospective studies comparing MA and MPA, alongside improved reporting of objective response and clinical benefit rates, would help further define their optimal use in modern practice. Future research should also explore predictive biomarkers of progestogen sensitivity to guide individualised therapy in late-line ER-positive metastatic breast cancer.

## Figures and Tables

**Table 1:** Baseline Clinical and Treatment Characteristics of the Study Cohort (N = 116).

Variable	N (%) or Median (Range)
Median age, years	69 (32–94)
Median ECOG Performance Status	2 (0–4)
Median prior lines of treatment	5 (1–11)
Median prior lines of endocrine therapy	3 (0–5)
Median prior lines of chemotherapy	3 (0–7)
Prior CDK4/6i exposure	
Yes	41 (35%)
No	75 (65%)
Histology	
IDC	87 (75%)
ILC	17 (15%)
Other	12 (10%)
Allred Score	
0–2	4 (4%)
3–5	3 (3%)
6–8	85 (73%)
Unknown	24 (20%)
Liver metastases	
Yes	86 (74%)
No	28 (24%)
Unknown	2 (2%)
Median duration of progestogen therapy, months	2.4 (0.1–26.4)

IDC, Invasive Ductal Carcinoma; ILC, Invasive Lobular Carcinoma; CDK4/6i, CDK4/6 inhibitor.

**Table 2:** Baseline Characteristics of Patients with Prolonged Disease Control (≥12 months).

Variable	Prolonged Disease Control	Rest of Cohort
Median age, years (range)	78 (65–88)	69 (32–94)
Median ECOG Performance Status (range)	2.5 (1–3)	2 (0–4)
Median prior lines of treatment (range)	4 (1–8)	5 (1–11)
Median prior lines of endocrine therapy (range)	3 (1–4)	3 (0–5)
Median prior lines of chemotherapy (range)	2 (0–5)	3 (0–7)
Prior CDK4/6i exposure	1/7 (14%)	41/112 (37%)
Histology		
IDC	4/7 (57%)	83/109 (76%)
ILC	2/7 (29%)	16/109 (15%)
Other	1/7 (14%)	10/109 (9%)
Liver metastases	3/7 (43%)	83/109 (76%)

IDC, Invasive Ductal Carcinoma; ILC, Invasive Lobular Carcinoma; CDK4/6i, CDK4/6 inhibitor.

**Table 3:** Reported Side-Effects of Oral Progestogens.

Reported Side-Effect	N (%)
Thromboembolic Events	10 (9)
Peripheral Oedema	13 (11)
Fatigue	9 (8)
Gastrointestinal Effects	9 (8)
Neurological (Headache/Dizziness)	8 (7)
Musculoskeletal Pain	5 (4)
Mood Changes	4 (3)
Hot Flushes	2 (2)
Menorrhagia	1 (1)

## Data Availability

The data presented in this study are not publicly available due to institutional governance restrictions and the inclusion of potentially identifiable patient information. De-identified data may be made available from the corresponding author upon reasonable request and subject to institutional approval and data sharing agreements, in accordance with applicable data protection regulations.

## Abbreviations

The following abbreviations are used in this manuscript:

MA	Megestrol Acetate
MPA	Medroxyprogesterone Acetate
SERD	Selective Oestrogen Receptor Degrader
ER-positive	Oestrogen Receptor-Positive
PFS	Progression-Free Survival
OS	Overall Survival
CI	Confidence Interval
HR	Hazard Ratio
AI	Aromatase Inhibitor
ECOG	Eastern Cooperative Oncology Group
CTCAE	Common Terminology Criteria for Adverse Events version 5.0
IDC	Invasive Ductal Carcinoma
ILC	Invasive Lobular Carcinoma
CDK4/6i	CDK4/6 inhibitor
